# Exploring Peri-Implantitis Risk-Factors: A Cross-Sectional Study

**DOI:** 10.3390/dj13040148

**Published:** 2025-03-28

**Authors:** Simina Angela Lăcrimioara Iușan, Ondine Patricia Lucaciu, Nausica Bianca Petrescu, Ioana Codruța Mirică, Dan-Alexandru Toc, Silviu Albu, Carmen Costache

**Affiliations:** 1 Department of Oral Health, Iuliu Hatieganu University of Medicine and Pharmacy, 400012 Cluj-Napoca, Romania; 2 Department of Microbiology, Iuliu Hatieganu University of Medicine and Pharmacy, 400012 Cluj-Napoca, Romania; 3 II-nd Department of Otolaryngology, Iuliu Hatieganudisx University of Medicine and Pharmacy, 400012 Cluj-Napoca, Romania

**Keywords:** peri-implantitis, dental implants, bacteria, microbiology, gender, smoking

## Abstract

**Background/Objectives**: With the increasing use of dental implants in edentulous patients and the high prevalence of peri-implantitis, understanding its microbial and risk factors is crucial. This study investigated Romanian patients from two private dental clinics in Cluj-Napoca, Romania, diagnosed with peri-implantitis, focusing on identifying the predominant bacterial species at affected sites compared with healthy implant sites. Additionally, we examined the impact of factors such as smoking, gender, age, and prosthetic restoration type on disease prevalence. **Methods**: This cross-sectional study, conducted between January 2023 and December 2024, included randomly selected patients who met the predefined inclusion and exclusion criteria. We enrolled 22 patients and 50 implants in the study. Data collected from medical records, clinical evaluations, and microbiological assessments were subsequently entered into a computerized database. Clinical data were analyzed using Social Science Statistics software(Jeremy Staangroom 2018). Bacterial samples were assessed, incubated, and subsequently identified using the Vitek 2 Compact System (BioMérieux, Marcy—l’ Étoile, France). **Results**: Peri-implantitis incidence was found to be independent of gender, more prevalent in the mandible, and equally affected smokers and non-smokers. The disease involves a complex polymicrobial infection, with pathogenic bacteria triggering the condition and opportunistic bacteria sustaining it. **Conclusions**: Peri-implantitis is a complex polymicrobial infection that arises from the interaction of strict pathogenic bacteria and opportunistic bacteria. Peri-implantitis results from intricate interactions of local, systemic, and microbial factors. Identifying its causes is essential for developing effective treatments, with future research emphasizing the role of opportunistic bacteria in disease progression.

## 1. Introduction

Oral health is a key indicator of overall health, well-being, and quality of life. Oral diseases pose a significant public health burden globally, affecting individuals throughout their lives. Despite advancements in living conditions and increasing urbanization, the prevalence of major oral diseases continues to rise. Tooth loss is often the result of long-term oral health issues, primarily severe periodontal disease or advanced dental caries, though it can also stem from trauma and other contributing factors [1]. A 2024 national study conducted by the College of Dentists in Romania concluded that about three out of four adults were identified with untreated edentations, three out of five had teeth loss as a result of carious lesions, and two out of five for other reasons [2]. The World Health Organization has also published a report on oral health in Romania, stating that in 2019, the prevalence of edentulism (complete loss of natural teeth) among individuals aged 20 and older was 12.4% [3]. Given these statistics, the need for oral rehabilitation among these patients is evident. Recent studies indicate that dental implants are becoming an increasingly common solution for replacing missing teeth among Romanian patients [4]. Research shows that the number of Romanians undergoing dental implant treatments tripled between 2013 and 2017 compared with the period from 2008 to 2012 [4]. This trend aligns with a market analysis report indicating that Romania’s dental implants market is expected to grow from USD 12.21 million in 2023 to USD 25.36 million by 2031. Between 2023–2031, the market is expected to expand at a compound annual growth rate of 9.7% [5]. Additionally, the global dental implants market was valued at USD 4.6 billion in 2022 and is projected to grow by 10% annually until 2030 [6]. Given this information and the rising popularity of dental implants, it is essential to be aware of potential complications that may arise from their use. In addition to issues like implant failure due to a lack of osseointegration or overload as well as the loosening or breakage of prosthetic components, dental implants can also be affected by inflammatory disorders impacting the surrounding tissues. These diseases are induced by peri-implant bacterial biofilms. There are two distinct pathologies: peri-implant mucositis and peri-implantitis. Peri-mucositis is known as an inflammatory lesion limited to the peri-implant mucosa, without ongoing marginal bone loss after bone remodeling. It is clinically identified by signs of inflammation such as bleeding on light probing, erythema, edema, and/or suppuration. Peri-implant mucositis is a reversible condition with the removal of the causative factor, bacterial biofilm. If left untreated, peri-mucositis can progress and potentially lead to peri-implantitis. Unlike peri-mucositis, peri-implantitis is characterized by inflammation of the peri-implant mucosa along with gradual bone loss [6]. Peri-implantitis is characterized by bleeding on probing, suppuration, increased probing depth, or recession of the gingival margin and radiographic bone loss [6]. The bacterial biofilm plays a crucial role in the initiation and progression of peri-implantitis by promoting chronic inflammation and facilitating the destruction of peri-implant bone [6,7]. There are some major risk factors for developing peri-implantitis such as poor plaque control, a history of severe periodontitis, and a lack of regular check-ups after implant placement [7]. The high prevalence of peri-implantitis and its potentially severe effects, such as implant loss, represents a major public health problem and involves substantial dental care costs [6].

Considering the aforementioned, we decided to investigate Romanian patients with peri-implantitis, diagnosed in two private dental clinics in Cluj-Napoca, Romania. The main purpose of our research was to identify the most common bacteria in peri-implantitis sites compared with healthy ones. Secondary objectives were to identify how smoking, gender, age of the patients, and type of the restauration influenced the primary characteristics of peri-implantitis and its frequency.

## 2. Materials and Methods

This cross-sectional study was conducted between January 2023 and December 2024 in two private dental clinics from Cluj-Napoca, with the data analyzed at the Department of Microbiology and Department of Oral Health of “Iuliu-Hatieganu” University of Medicine and Pharmacy, Cluj-Napoca. The study adhered to the criteria specified in the Declaration of Helsinki and was approved by the Ethics committee (number: AVZ 9/06 January 2023) of “Iuliu-Hatieganu” University of Medicine and Pharmacy.

### 2.1. Study Sample

The subjects in the current study were randomly selected from patients from two clinics across the Cluj region and consecutively enrolled in the study in accordance with the inclusion and exclusion criteria listed below. All study participants filled out a series of forms and gave their informed consent.

Given the exploratory nature of this study, our primary objective was to gather preliminary data that could later serve as a basis for estimating sample sizes in future research. The determination of an appropriate sample size relies on estimates of key parameters, such as standard deviation and success rate, as well as epidemiological data on disease prevalence at the population level. However, in Romania, centralized data on peri-implantitis patients are not available. In the absence of prior studies or relevant preliminary data, an accurate calculation of the required sample size was not feasible; therefore, the sample size was determined empirically.

### 2.2. Eligibility Requirements

Participants were selected for the study in accordance with the following inclusion criteria: patients between 18 and 80 years of age, males and females, patients with at least one missing tooth and restored with an implant-supported prosthesis, smoking (at least one cigarette per day) and non-smoking (specifically, never a smoker), at least one year following implant insertion.

Patients with the following conditions were excluded: uncontrolled systemic disease, pterygoid and zygomatic implants, pregnant patients, and those who had taken antibiotics within the previous two weeks of the examination.

### 2.3. Case Definitions

Healthy sites were diagnosed using case definition criteria according to clinical practice guidelines [6] including the absence of inflammation signs, no bleeding or suppuration during probing, and no bone loss after remodeling. Peri-implantitis was diagnosed based on the same guidelines: presence of bleeding and/or suppuration, presence of bone loss beyond the initial remodeling, and probing depth > 6 mm [6].

### 2.4. Outcomes

Primary outcome: microbiological results.

Secondary outcomes: edema, redness, bleeding and suppuration at gentle probing, pus presence, marginal bone loss.

### 2.5. Data Acquisition

The information was collected based on medical files, clinical and microbiological examinations, and was transferred to a computerized database.

#### 2.5.1. Medical Files

Medical files were used to collect information on age, gender, medical history of periodontitis, implant location (maxillary/mandibular), time of implant insertion/implant uncover, brand of implant, type of prosthesis (screwed or cemented), smoking habit, and presence of general health issues.

#### 2.5.2. Clinical Examination

A single calibrated examiner (S.A.L.I.) performed the clinical measurements at six different sites per implant and registered clinical information such as edema, redness, bleeding and suppuration at gentle probing, pus presence, marginal bone loss. The presence of bleeding on gentle probing was determined using a manual periodontal probe placed circumferentially into the peri-implant sulcus with 0.2 N pressure. If bleeding occurred in less than 30 s, the site was considered bleeding on probing positive. Using a periodontal probe, the examiner also measured the peri-implant probing depths circumferentially at six sites and recorded the higher value per implant.

### 2.6. Microbiological Samples Collection and Examination

The implants selected for sampling were isolated using cotton rolls and saliva aspiration. The working area was dried with air spray and the marginal plaque was removed with sterile compresses. Following the evaluation of clinical outcomes and measures, microbiological samples were collected by inserting six sterile endodontic paper points into each peri-implant sulcus (mesio-buccal, central-buccal, disto-buccal, mesio-lingual, central-lingual, disto-lingual) twice for 30 s. These paper points were placed in two separate transport medium recipients: one for aerobic, anaerobic, and fastidious bacteria (Liquid Amies Medium, Liquid Based Collection, and Transport 482C, Copan Brescia, Italy, respectively) and one for facultative and obligate anaerobic bacteria (Thioglycolate Broth Medium 4U002N, Copan USA). The microbiological probes were stored at room temperature and delivered to the microbiology laboratory to be analyzed within 24 h, as indicated by the manufacturer. At the laboratory, the samples were cultivated using a 10 μL inoculation loop (for quantitative measure of the total bacterial count) on Columbia Agar with 5% sheep blood (BioMérieux, Marcy—l’ Étoile, France) and on Brucella Blood Agar (Thermo Fisher Scientific, Waltham, MA, USA). The plates were further incubated for 24 h at 37 °C in aerobic conditions and for 48 h at 37 °C in anaerobic conditions. The bacterial cultures were analyzed, and we established a minimum threshold of 10 colonies with the same morphology (corresponding to the 10^3^ CFU/mL) that were replated, incubated, and then diagnosed using the Vitek 2 Compact System (BioMérieux, Marcy—l’Étoile, France). Based on the total number of colonies with the same morphology, three groups were formed: 10^3^–10^4^ CFU/mL (between 10 and 100 colonies), 10^4^–10^5^ CFU/mL (between 100 and 1000 colonies), and >10^5^ CFU/mL (more than 1000 colonies). This approach will provide insights into the total bacterial burden for positive samples and will facilitate the differentiation between contaminants and true pathogens.

### 2.7. Reproducibility and Repeatability

One examiner (S.A.L.I.) realized all of the measurements and collected the microbiological probes for the investigation. To ensure reproducibility of the results, the examiner was calibrated with an experienced co-author (O.P.L.) who served as a reference. Both investigated five implants from individuals who were not part of the study. The reproducibility of the results was assessed by comparing the results. The two examiners agreed that if there were any disparities in measurements, they would re-examine the implant together to determine the proper value. There were no disagreements between the two examiners.

### 2.8. Statistical Analysis

Results were stored in a Microsoft Excel database. All data were analyzed using Social Science Statistics software [8]. We used the chi-square test with Yates’ correction for small numbers for categorical variables (number of peri-implantitis on maxilla and mandibula, male/female, smokers/non-smokers, periodontal disease/no. of implants with peri-implantitis in gender and location), Mann–Whitney U test, and the Student’s *t*-test for independent variables to analyze the differences between groups (probing depth, peri-implantitis occurrence interval). Dataset normality was tested with the Shapiro–Wilk test. Statistical significance was set at *p* < 0.05 [8].

### 2.9. Risk of Bias Assessment

To minimize potential sources of bias in our study, we carefully controlled several key variables. First, we selected patients exclusively from two clinics and limited the surgical procedures to only two experienced surgeons. This approach aimed to reduce the risk of peri-implantitis caused by surgical errors. Additionally, we included only three different implant systems to prevent subjective assessments related to implant brand preference. Furthermore, all clinical measurements were conducted by a single evaluator, thereby eliminating inter-observer variability and ensuring consistency in data collection. These measures were implemented to enhance the reliability and validity of our findings.

## 3. Results

### 3.1. Study Population

Thirty patients treated with dental implants were examined for eligibility based on the inclusion and exclusion criteria. Twenty-two patients (50% males, 50% females, mean age 49.2, SD = 10.05) met the necessary requirements and were enrolled in the clinical study. A total number of fifty implants were examined (26 with peri-implantitis and 24 without peri-implantitis) and recorded (Table 1).

### 3.2. Clinical Outcomes

We found significant statistical differences between groups (*p* = 0.01101, χ^2^ = 6.46). There were correlations between the 16 patients with peri-implantitis and the following symptoms in the larger sample of 22 patients. Those with peri-implantitis exhibited bone loss, edema, redness, bleeding, suppuration, and pus compared with those without peri-implantitis (Table 2). Out of the patients enrolled in this study, 16 patients had 26 implants with peri-implantitis and 11 healthy implants, and the other 6 patients had 13 healthy implants and 0 implants with peri-implantitis (Table 1).

#### 3.2.1. Gender Distribution

In terms of gender distribution, both lots were balanced. We enrolled 11 males and 11 females in this study. Each gender had eight people with peri-implantitis (72.7%) (Table 3).

**Figure 1:**
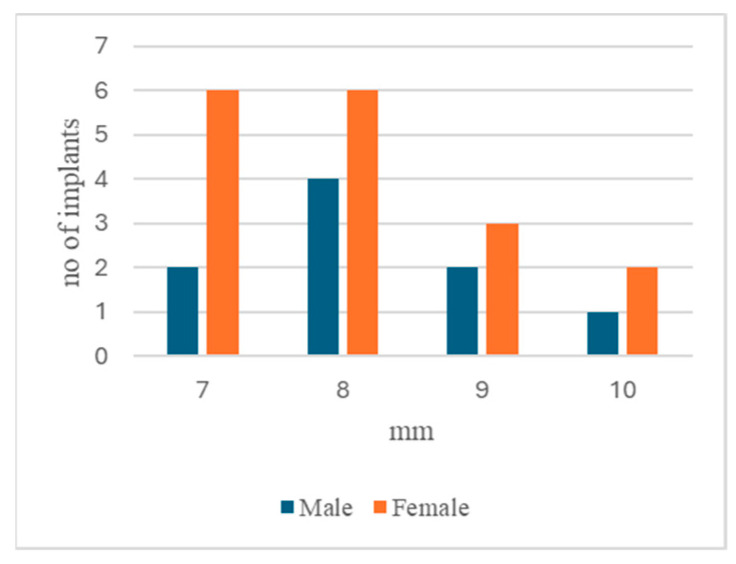
Probing depth distribution between males and females.

In both groups, for men and women who had a history of periodontal disease, the number of implants affected by peri-implantitis was 50%. The time interval after which peri-implantitis occurred after implant insertion was much longer in males (with an average of 11.87 years for men and 6.11 years for women, *p* = 0.0055) but there was no significant difference in the probing depths for males and females (Figure 1). Peri-implantitis was approximately equally frequent in males and females. The only significant distinction was the fact that men were more likely to develop pus: the men had nine implants with peri-implantitis and five implants with pus while the women had seventeen implants with peri-implantitis and three implants with pus (*p* = 0.046322, χ^2^ = 3.9699) (Table 3 and Table 4).

#### 3.2.2. Age Groups

Among the three age groups mentioned in Table 5 and Table 6, no significant statistical differences were obtained. Somewhere at the limit of statistical significance was the number of cases of peri-implantitis, respectively 100% for those over 60 years old, but there were only two patients. The under forty and over sixty age groups were too small for analysis. The probing depths had close average values as well as peri-implantitis occurrence time intervals, but without statistically significant differences (Table 5 and Table 6).

#### 3.2.3. Smoker/Non-Smoker Distribution

In our patient population, peri-implantitis affected both smokers and non-smokers in roughly comparable percentages (66.7% smokers vs. 69.2% non-smokers) (Table 7).

**Figure 2:**
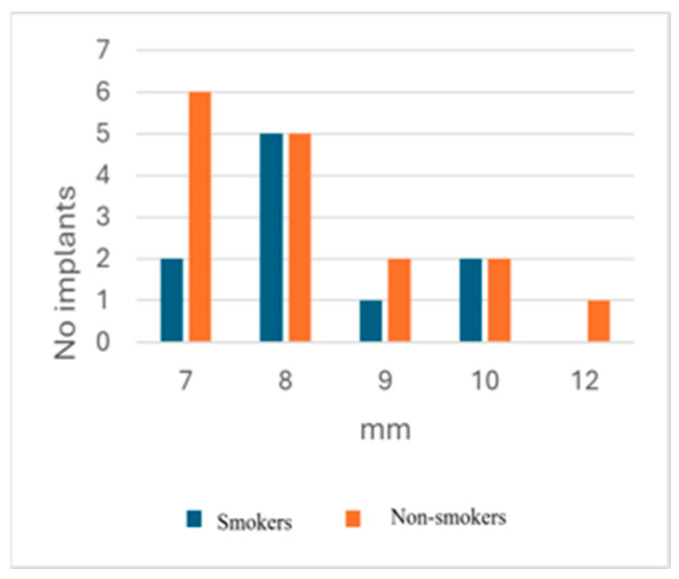
Probing depth distribution between the smokers and non-smokers.

In our groups, smokers had peri-implantitis at the level of the mandible, while non-smokers had four patients with peri-implantitis at the maxillary level and five at the mandible level. According to our data, edema, redness, BoP, suppuration, and pus were more frequent in patients from the non-smoking group compared with those in the smoking group (Table 7). The mean probing depth values were close for the smoking/non-smoking groups (8.3 mm for smokers, 8.25 mm for non-smokers) (Table 8) (Figure 2). The peri-implantitis occurrence interval was shorter for smokers in comparison with the values of non-smokers (Table 8). The values of the peri-implantitis occurrence interval were very scattered in both groups.

The six smokers with peri-implantitis had ten implants: two of them with edema and two with redness, while the nine non-smokers with peri-implantitis had sixteen implants: thirteen of them with edema and thirteen with redness. Therefore, for both edema and redness, there was a statistical correlation: χ^2^ = 7.1155, *p* = 0.007642.

#### 3.2.4. Maxilla/Mandibula Distribution

The mandible was more rapidly and frequently affected by peri-implantitis than the maxilla, with a statistical significance (*p* = 0.01101, χ^2^ = 6.46); the remaining symptoms related to peri-implantitis were not significantly different between the maxilla and the mandible (Table 9).

Table 9 and Table 10 shows that the mandible was more frequently and more quickly affected by peri-implantitis compared with the maxilla.

**Figure 3:**
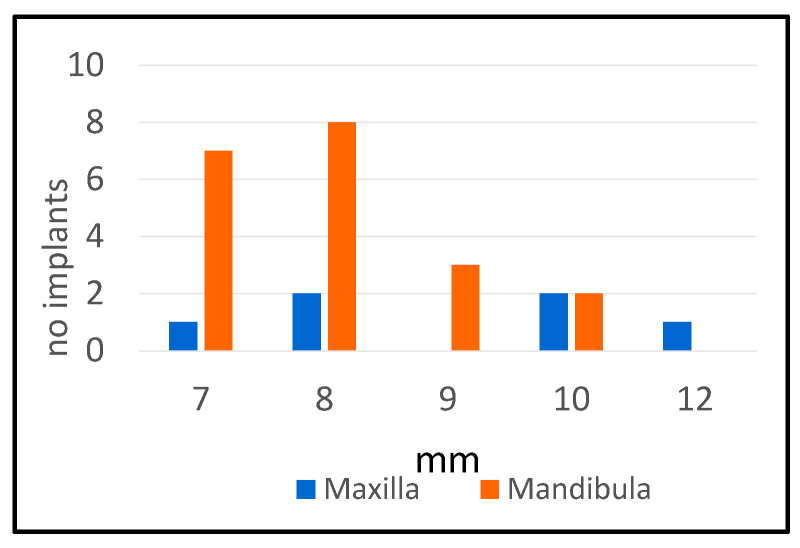
Probing depth distribution.

Probing depths in cases with peri-implantitis ranged from 7 to 12 mm (Figure 3). The average value of the probing depth was higher in the maxilla, and the time interval after which peri-implantitis occurred was shorter in the mandible, with statistically significant differences (Table 9).

Of those with a history of periodontal disease, the following implants developed peri-implantitis: 40% (two out of five) on the maxilla and 54.55% (six out of eleven) on the mandible, with no statistically significant difference.

#### 3.2.5. Stage and Type of Prothesis

Regarding the type and stage of the prosthetic work at the time of the study, we had the following situation among our patients: 32 implants with final prosthetic restoration (16 with cemented restoration, 12 with screwed restoration, and 4 with removable overdenture), 15 implants with temporary restoration made from PMMA (polymethyl methacrylate), and 3 implants with healing screws (Table 11).

The highest incidence of peri-implantitis (68,75%) was recorded for the fixed cemented prosthesis and the deepest probing depths (9.09 mm) when compared with the screwed fixed prosthesis and the screwed fixed prosthesis constructed of PMMA. There was a statistically significant association between peri-implantitis and cemented-retained crowns (Table 11 and Table 12).

Two types of implants were used more frequently: Megagen AnyOne and Megagen AnyRidge. It is noteworthy that while in the case of Megagen AnyOne, peri-implantitis occurred in 75% of the implants (9 out of 12), in the case of Megagen AnyRidge (8 out of 24), it only occurred in 33.34%, the difference being statistically significant (χ^2^ = 6.6728, *p* = 0.018242).

The average probing depths were similar, 8.11 mm vs. 7.62 mm, and the peri-implantitis occurrence interval was longer for Megagen AnyOne (5.66 years vs. 3.35 years), but the difference was not statistically significant. (Table 13).

In relation to other conditions, three patients had hypertension, and two of them also had peri-implantitis, one with a cemented fixed denture, and the other with a screwed PMMA prosthesis. There were two patients diagnosed with diabetes, both with peri-implantitis and an average probing depth of 8.2 mm, which was comparable to that of the peri-implantitis patients.

### 3.3. Microbiological Results

Bacterial identification revealed that bacteria occurrence was more frequent in cases of implants affected by peri-implantitis (Table 14).

The bacteria present in more than 10^5^ CFU/mL were found mostly in peri-implantitis sites: *Kocuria rosea*, *Leuconostoc mesenteroides* ssp. *cremoris*, *Streptococcus gordonii*, *Streptococcus constelatus*, *Streptococcus pseudoporcinus*, *Enterococcus faecalis*, *Yokenella regensburgei*, and *Staphylococcus aureus*. The following bacteria were identified in peri-implantitis sites with 10^4^–10^5^ CFU/mL: *Streptococcus anginosus*, *Enterococcus faecalis*, *Streptococcus gordonii*, *Leuconostoc mesenteroides* ssp. *cremoris*, *Enterococcus faecalis*, *Kocuria rosea*, and *Streptococcus gordonii.* There were sites with peri-implantitis where bacteria were present in only 10^3^–10^4^ CFU/mL: *Kocuria rosea*, *Leuconostoc mesenteroides* ssp. *cremoris*, *Staphylococcus epidermidis*, *Staphylococcus aureus*, and *Pseudomonas aeruginosa*.

Regarding implants with healthy sites, we found: *Staphylococcus epidermidis* and *Staphylococcus hominis* ssp. *hominis* in three sites with 10^5^ CFU/mL; *Staphylococcus hominis* ssp. *hominis*, *Enterococcus fecalis*, *Streptococcus intermedius,* and *Sphingomonas paucimobilis* in four sites with 10^4^–10^5^ CFU/mL; and *Streptococcus oralis*, *Streptococcus sanguinis*, *Staphylococcus epidermidis*, *Staphylococcus hominis* ssp. *hominis*, *Streptococcus intermedius*, and *Enterococcus faecalis* in six sites with 10^3^–10^4^ CFU/mL (Table 15).

Regarding the bacterial distribution by genus, the most frequently encountered in our samples were bacteria from the genus *Streptococcus*, followed by *Staphylococcus* and *Enterococcus* (Table 16).

## 4. Discussion

### 4.1. Principal Findings—Consistency and Discrepancies with Previous Findings

Based on our research, all of the implants with peri-implantitis exhibited bone loss with probing depths between 7 and 12 mm. Bleeding on probing was present in 88.46% of implants, 84.62% presented suppuration, 58% redness and edema, and 30.77% experienced pus. There were no healthy implants with these manifestations. These results suggest a positive correlation between these symptoms and peri-implantitis (*p* < 0.05). According to the EFP S3 level clinical practice guidelines published recently in 2023 [6], peri-implantitis exhibits the presence of bleeding on probing and/or suppuration on gentle probing and probing depths ≥ 6 mm, which is in accordance with our results.

Our study groups were calibrated in terms of gender distribution. Both groups, male and female, had an equal number of patients and the same percentage of peri-implantitis, so we can state that gender does not influence the occurrence of peri-implantitis. Some studies have suggested a significant association between peri-implantitis and male gender [9,10,11,12], while others did not find any correlation between peri-implantitis and gender [13,14].

In terms of smoking habits, peri-implantitis affected both smokers and non-smokers in comparable percentages. This finding suggests that smoking is not a risk factor for peri-implantitis, which is in agreement with other studies [10,13,15]. There are also published studies that suggest that smoking is a risk factor for peri-implantitis occurrence related to an increased prevalence of peri-implantitis [11,12,14]. The result of the present study could be influenced by the small number of participants and/or by the inequality between the two groups, smokers and non-smokers, and by the criteria used to consider the subject as a smoker (at least one cigarette/day). According to our findings, edema, redness, bleeding on probing, suppuration, and pus are more prevalent in patients from the non-smoking group than in the smoking group. This is consistent with earlier research that indicates that smoking and nicotine from cigarettes alter the gingival epithelium. Smoking reduces inflammatory cell infiltration and blood vessel density among gingival tissue. In the tissues of smokers, symptoms of inflammation are suppressed in the early stages, which is also translated into a low BoP index [16,17]. This effect creates difficulties in diagnosing the disease in the early stages. Light symptoms of inflammation are suppressed by smoking, but bone loss and pocket formation start and progress [16,17].

We did not find a statistically significant correlation between the history of periodontal disease and peri-implantitis. There has been previous research that underlies the association between the history of periodontitis and peri-implantitis [10,18,19], but our results may differ due to the limited number of patients affected by periodontitis in our groups.

Our study indicates a positive correlation between peri-implantitis and cemented permanent implant supported dentures, which is consistent with the findings of previous studies [11,12,14,15,19] that suggest a correlation between cement-retained crowns and peri-implantitis and also emphasize that more implants with cemented prostheses are affected by peri-implantitis than those screw-retained.

Microbial identification revealed a large variety of microorganisms into the peri-implantitis sites and few in healthy sites. There were three healthy sites in which *Staphylococcus epidermidis* (two sites) and *Staphylococcus hominis* ssp. *hominis* (one site) had a bacterial density higher than 10^5^ CFU/mL. Both are coagulase-negative staphylococci, commonly found as part of the human skin and mucosal microbiota. While typically non-pathogenic, they can act as opportunistic pathogens in specific conditions, particularly in immunocompromised individuals or when associated with medical devices including dental implants [20,21]. *Staphylococcus hominis* ssp. *hominis* was also identified in two healthy sites with a bacterial density of 10⁴–10⁵ CFU/mL, and in one site with a density of 10^3^–10⁴ CFU/mL.

In healthy sites, we found also *Streptococcus intermedius* in one place with a density of 10⁴–10⁵ CFU/mL and in another place with a density of 10^3^–10⁴ CFU/mL. *Streptococcus intermedius* is a facultative anaerobic, Gram-positive coccus, commonly found in the oral cavity, gastrointestinal tract, and upper respiratory tract as part of the normal microbiota. Despite being a commensal microorganism, it is also an opportunistic pathogen causing invasive suppurative infections [22].

*Streptococcus oralis* is a component of the normal human oral microbiome with opportunistic pathogenicity [23]. This was isolated from one healthy site with a low bacterial density of 10^3^–10⁴ CFU/mL.

There were also two healthy sites from which *Enterococcus faecalis* was isolated, a Gram-positive, facultatively anaerobic bacteria. Normally a commensal of the human gastro-intestinal tract, *Enterococcus faecalis* is also an opportunistic pathogen known for its role in various infections, particularly in biofilm-associated conditions [24].

*Sphingomonas paucimobilis* is a Gram-negative, aerobic rod-shaped bacterium from the *Sphingomonas genus. S. paucimobilis* is typically found in soil, water, and biofilms in hospital environments such as water systems and medical equipment. Its presence in people is usually temporary and is caused by environmental exposure (for example, contaminated water or hospital facilities), but it can also colonize and cause opportunistic infections, particularly in immunocompromised individuals or through contaminated medical equipment [25].

Regarding the sites affected by peri-implantitis, most microorganisms isolated from those sites were with a high bacterial density, higher than 10⁵ CFU/mL. Of these, *Enterococcus faecalis* was the most frequent, found around four implants with peri-implantitis. We also isolated *E. faecalis* from two sites with a bacterial density of 10^4^–10^5^ CFU/mL. As we previously specified, *Enterococcus faecalis*, despite being a commensal bacterium, is also an opportunistic pathogen and, as mentioned in other studies, could be a specific pathogen for peri-implantitis, which is consistent with our findings [26,27].

There were two sites in which *Streptococcus pseudoporcinus* was found with a high bacterial density. *Streptococcus pseudoporcinus* is a beta-hemolytic Gram-positive non-motile coccus first isolated in 2006 from the genito-urinary tract of women. Although it has not been well-studied, some studies have reported serious infections caused by this bacterium [28,29,30]. Taking into consideration its high potential to cause infections, we cannot exclude the possibility of being involved in peri-implantitis pathogenicity in specific conditions. At this moment, we did not find any studies on this subject; only a study that mentioned that this bacterium was found in a healthy peri-implant situs [31].

Other bacteria from *Streptococcus genus* found in peri-implantitis sites were *Streptococcus gordonii* and *Streptococcus constellatus*. *Streptococcus gordonii*, having an increased bacterial density of 10⁴–10⁵ CFU/mL and higher, is a Gram-positive commensal microorganism found on the skin, in the mouth cavity, and intestine. It is also known as a pathogenic opportunistic bacterium that can cause localized or systemic conditions [32]. It is an early colonizer on implant surfaces, facilitating the adherence and growth of more destructive bacteria [33]. It has been demonstrated that *Streptococcus gordonii* can provide a scaffold for more pathogenic bacteria, like *Porphyromonas gingivalis*, in oral plaque. Later colonizers, such as *P. gingivalis*, require the presence of early colonizers to adhere and grow within the biofilm. Studies have revealed that *S. gordonii* physically co-aggregates with *P. gingivalis* through particular protein–protein interactions [34]. *Porphyromonas gingivalis,* a Gram-negative anaerobic bacterium and member of the red complex, which is highly pathogenic and contains particularly aggressive bacterium species, is known as an important pathogen involved in the development of peri-implantitis [35]. This information provides evidence for *S. gordonii*’s role in the occurrence and development of peri-implantitis.

Our findings regarding the presence of *Streptococcus constellatus* in peri-implantitis sites are in accordance with other studies [35,36,37]. *Streptococcus constellatus,* a member of the *Streptococcus milleri* group, is a commensal bacteria found in the oral cavity and upper respiratory tract. Despite it belonging to the normal flora, *S. constellatus* is an opportunistic bacterium, a significant contributor to tissue infections characterized by tissue destruction and suppuration. A member of the orange complex, *S. constellatus* is a pathogen of peri-implantitis, and its role in abscess and suppuration formation may exacerbate severe cases, especially those involving mixed anaerobic flora [35,38,39].

Another bacterium present in peri-implantitis sites is *Staphylococcus aureus.* An opportunistic Gram-positive bacterium, normally found on skin and mucous membranes, *Staphylococcus aureus* is also an important human pathogen that can cause a variety of conditions, from minor skin disorders to serious systemic infections [40]. Numerous studies have reported the influence of *S. aureus* in the pathogenesis of peri-implantitis. Well-known for its significant capacity to adhere to almost any titanium biofilm [37,41], *S. aureus*, an early colonizing microorganism [42,43], seems to have an important role in the occurrence of peri-implantitis [37,43,44,45,46].

*Kocuria rosea* is another bacterium isolated from peri-implantitis affected implants in high bacterial density (>10^5^ CFU/mL) from two sites and medium bacterial density (10^4^–10^5^) at one site. Human skin, mucosae, and the oral cavity are typically colonized by these commensal bacteria [47]. It is generally considered a harmless environmental and commensal microorganism but can act as an opportunistic pathogen under specific conditions. There have been reported cases in which it was involved in infections associated with medical devices [47,48,49], so we cannot exclude the possibility of it being associated with peri-implantitis development in specific cases.

Another two species, *Leuconostoc mesenteroides* ssp. *cremoris* and *Yokenella regensburgei*, were cultivated from peri-implantitis sites. *Leuconostoc mesenteroides* ssp. *cremoris* was found with increased bacterial density >10^5^ CFU/mL, medium 10⁴–10⁵ CFU/mL, and low-density. *Leuconostoc mesenteroides* ssp. *cremoris* is an obligate heterolactic fermentative lactic acid bacterium that is primarily used in industrial dairy fermentation [50]. This bacterium could colonize the oral cavity due to the consumption of foods and vegetables but is also recognized as a potential pathogen. Some studies have mentioned the role of this bacterium in various infections in humans, and one specified the implication of *Leuconostoc mesenteroides* in the occurrence of an odontogenic infection [51,52,53,54].

Due to the presence of this pathogenic strain of *Leuconostoc mesenteroides*, we cannot exclude its potential pathogenic role in peri-implantitis. The dual behavior of *Leuconostoc mesenteroides* emphasizes the need for additional research to better understand this bacterium’s biology.

*Yokenella regensburgei*, a Gram-negative, oxidase-negative rod, was initially reported by Kosako et al. in 1984 [55]. It is the only species of the *genus Yokenella* and belongs to the *Enterobacteriaceae family. Y. regensburgei* is rarely isolated from people, hence its clinical significance is unknown. There are documented cases of this bacterium causing infections in various body sites [56,57,58], but to our knowledge, at this moment, there are no reported cases of oral infections caused by *Yokenella regensburgei.* We isolated *Yokenella regensburgei* (bacterial density > 10^5^ CFU/mL) from a peri-implantitis site in a young patient with a history of periodontitis and no general health issues. Further studies are needed to evaluate the pathogenic potential of this bacterium in peri-implantitis.

In one peri-implantitis situs, we isolated *Streptococcus anginosus* (10⁴–10⁵ CFU/mL). *Streptococcus anginosus* is a species of bacteria that is part of the *Streptococcus genus*. It is considered a member of the *Streptococcus milleri* group, which includes several other related species like *Streptococcus constellatus* and *Streptococcus intermedius*. *Streptococcus anginosus* is part of the normal flora of the human body, especially in the oral cavity, upper respiratory tract, and gastrointestinal tract. Although it is part of the normal microbiota, *Streptococcus anginosus* can become pathogenic and cause infections [59]. Research data consistent with our findings also revealed the presence of *Streptococcus anginosus* in the pathogenesis of peri-implantitis [36,45]. Normally a commensal bacterium, we cannot ignore the opportunistic behavior of this pathogen and its potential to form biofilms [36] as mechanisms for increasing the likelihood of microbial interactions and the development of peri-implantitis.

*Staphylococcus epidermidis* was mostly isolated from healthy implants and from one situs of peri-implantitis (10^3^–10^4^ CFU/mL). *Staphylococci* are ubiquitous bacteria that colonize the skin and mucous membranes of humans and other mammals [60]. Usually, a commensal bacterium, *Staphylococcus epidermidis* has also been mentioned in other studies, being occasionally isolated from peri-implantitis sites, knowing its affinity for medical devices [44,45]. *Staphylococcus epidermidis* could have a probiotic function, acting as a natural competitor to more harmful bacteria, such as *Staphylococcus aureus*, and maintaining the balance of the local microbiome [60]. Our findings are consistent with those existing in the literature, and the presence of *Staphylococcus epidermidis* mainly in healthy sites proves its low pathogenicity and commensal role.

In one case of peri-implantitis, we isolated *Pseudomonas aeruginosa*, a Gram-negative rod frequently found in soil, water, and dentistry water lines as well as in the mouths of hospitalized patients [61]. Some studies have detected this bacterium near failed dental implants and in peri-implantitis areas [43,61,62]. Research indicates that *P. aeruginosa*, aside from its challenging eradication when structured in a biofilm, exhibits resistance for more than 80% of the antibiotics evaluated [27]. This suggests that the antimicrobial resistance of opportunistic pathogens in peri-implantitis necessitates the urgent development of alternative treatments to antibiotics for managing these infections [27].

While commensal bacteria typically maintain a balanced, non-pathogenic relationship with their host, a phenomenon known as eubiosis, disruptions in the oral microbiome or host immune system can turn these otherwise harmless microbes into contributors to peri-implantitis. These conditions can lead to peri-implant illnesses caused by opportunistic and pathogenic bacteria in dysbiosis. Gram-negative anaerobic pathogens and opportunistic microorganisms appear to be the primary contributors behind peri-implantitis. By activating their virulence factors, such as secreting endo-/exo-toxins and enzymes, certain microbes can change from commensal to pathogenic, causing in situ tissue destruction. Commensals can also interact with established pathogens (e.g., *Porphyromonas gingivalis* and *Fusobacterium nucleatum*) to enhance their virulence [27,63,64]. Aggressive pathogens from the red and orange complex, along with other pathogenic species (*P. gingivalis, T. denticola, T. forsythia, P. intermedia, C. rectus*, etc.), have been extensively studied and associated with the initiation and progression of peri-implantitis in numerous studies [35,65,66,67]. Our study identified facultative anaerobic, opportunistic, and potentially pathogenic bacterial species at peri-implantitis sites, which have been less studied but may contribute to disease progression and severity by influencing the local microbiome.

Peri-implant disease appears to be initiated and maintained in large part by the presence of a biofilm and factors that promote plaque retention surrounding an implant [44,63]. Oral biofilm, a three-dimensional structure made up of several communities of bacteria embedded in an extracellular matrix, can be formed by oral microorganisms [64].

The bacterial biofilm is a complex structure developed in several consecutive steps. Initially, bacterial cells adhere to the surface and start developing microcolonies. The next step is developing a mature biofilm with an intricate extracellular matrix, extracellular DNA, and other molecules. The final step of a bacterial biofilm is dispersal [68,69].

With such an elaborate mechanism of development and structure, it is of no surprise that this structure is involved in several types of infections including peri-implantitis [6,70]. Unfortunately, the development of a bacterial biofilm implies several challenges in eradicating an infection caused by it, specifically the antimicrobial resistance it may cause and the resistance to antiseptics and other substances. The involvement of bacterial biofilms in human oral infections has been evaluated in several previous studies and it may be a future target for personalized oral treatments [68,71,72,73].

Peri-implantitis is a complex inflammatory condition that affects the tissues surrounding dental implants, leading to bone loss and potential implant failure. Currently, despite many surgical and non-surgical treatment options, there is no single curative treatment that guarantees a complete resolution in all cases. However, different treatment modalities have been developed to manage and control the progression of the disease. Treatment of this condition is quite challenging due to the existence of several risk factors, a variety of surfaces that are difficult to disinfect, and the formation of bacterial biofilms that are hard to remove mechanically and are challenging to reach with medication therapies [74,75,76,77,78].

Given the increasingly widespread use of dental implants, it is important to understand the pathogenic mechanisms of peri-implantitis, a condition that poses a concern for dentistry due to its potential to compromise the long-term success of dental implant treatments. The complexity of this condition arises from the interaction between multiple local and systemic risk factors as well as microbial factors. Recognizing the etiological factors that can lead to the onset and progression of this condition is crucial for the development of new and more effective therapeutic approaches. To create successful therapeutic strategies for the eradication of peri-implantitis, future research could focus on the pathogenic potential of opportunistic commensal bacteria and how these interact with pathogenic bacteria in peri-implantitis to increase their virulence.

The findings of this study may be generalizable to other populations with comparable clinical characteristics, treatment protocols, and implant systems. However, caution is warranted when extending these results to broader populations, as variations in demographic factors, surgical techniques, and oral health conditions could influence the observed outcomes.

### 4.2. Limitations of the Research

Several limitations of the research should be discussed. One limitation of this study was the relatively small sample size, consisting of 22 patients and 50 implants. Although the cross-sectional design allowed for the identification of associations between peri-implantitis and various factors, the limited number of participants may affect the generalizability of the findings. A small sample also increases the risk of selection bias, as the studied population may not fully represent broader clinical cases. Additionally, the statistical power of the analysis is reduced, potentially underestimating the true associations between risk factors and peri-implantitis. Furthermore, the small sample size limits the ability to perform robust subgroup analyses, which could provide deeper insights into variations in implant characteristics and patient-related factors. Future studies with larger and more diverse patient cohorts are recommended to enhance the reliability and applicability of the results.

In addition, information on smoking status and general health status was self-reported by the patients; as a result, variations regarding the actual tobacco exposure and health problems are likely. Another limitation was the criteria used to identify a smoker (one cigarette per day) and the absence of a smoking classification (mild, moderate, or heavy smoker). Another concern was related to the microbiological examination of the probes, which were unable to discover strictly anaerobic bacteria due to technological limitations.

## 5. Conclusions

Summarizing the results, we can conclude that peri-implantitis is a complex polymicrobial infection resulting from the interaction of strictly pathogenic bacteria, which are capable of initiating and sustaining disease independently, and opportunistic bacteria, which exploit favorable conditions, such as host immune dysregulation or biofilm formation, to contribute to the progression of the infection. The incidence of peri-implantitis appears to be independent of gender, occurs more frequently in the mandible than in the maxilla, and affects smokers and non-smokers in approximately equal proportions.

Our study underlies the presence of opportunistic pathogens in peri-implantitis onset, and further clinical studies should be developed to find new treatments against these bacterial interactions and the development of peri-implantitis.

## Figures and Tables

**Table 1:** Distribution of the patients and implants.

	Patient No.	Implant No.	
Total	22	50	
		Implants with Peri-Implantitis	Healthy Implants
Patients with peri-implantitis	16	26	11
Patients with healthy implants	6	0	13

**Table 2:** Correlations in the general group between those who had peri-implantitis and the rest of the symptoms.

Condition	No. of Implants		Percentage
	Yes	No	
Peri-implantitis	26	24	
Bone loss	26	0	100%
Edema	15	0	58%
Redness	15	0	58%
Bleeding	23	0	88.46%
Suppuration	22	0	84.62%
Pus	8	0	30.77%

**Table 3:** Symptom distribution among genders.

	Male	%	Female	%
No. of patients	11		11	
No. of patients with peri-implantitis	8	72.7%	8	72.7%
Vertical bone loss	8	100%	8	100%
Edema	5	62.5%	5	62.5%
Redness	6	75%	4	50%
Bleeding	7	87.5%	7	87.5%
Suppuration	6	75%	6	75%
Pus	4	50%	2	25%
Bacteria occurrence	7	87.5%	4	50%

**Table 4:** Gender average probing depths and the peri-implantitis occurrence interval for male/female implants.

	Average Probing Depths mm ± SD	Peri-Implantitis Occurrence Interval Years ± SD	No. of Implants with Peri-Implantitis/Pus
Male	8.67 ± 1.65	11.87 ± 3.6	5 out of 9
		t = 2.8953, *p* = 0.0055	χ^2^ = 3.9699, *p* = 0.046322
Female	8.06 ± 1.03	6.11 ± 4. 48	3 out of 17

**Table 5:** Symptom distribution among age groups.

Age Group	<40 Years	%	40–59 Years	%	≥60 Years	%
No. of patients	6		14		2	
No. of patients with peri-implantitis	3	50%	9	64.3%	2	100%
Vertical bone loss	3	100%	9	100%	2	100%
Edema	3	100%	4	44.5%	1	50%
Redness	2	66.7%	5	55.5%	1	50%
Bleeding	3	50%	7	77.7%	2	100%
Suppuration	2	66.7%	6	66.7%	2	100%
Pus	1	33.4%	1	11.1%	1	50%
Bacteria occurrence	2	66.7%	7	77.7%	1	50%

**Table 6:** The average probing depths and peri-implantitis occurrence interval for implants in age groups.

	Average Probing Depths mm ± SD	Peri-Implantitis Occurrence Interval Years ± SD
<40 years	8.33 ± 0.577	7± 2.04
40–59 years	8.11 ± 1,31	9.25 ± 4.78
≥60 years	8.8 ± 2.05	6 ± 4.42

**Table 7:** Symptom distribution among the smokers and non-smokers.

	Smokers	%	Non-Smokers	%
No. of patients	9		13	
No. of patients with peri-implantitis	6	66.7%	9	69.2%
Vertical bone loss	6	100%	9	100%
Edema	2	33.4%	8	88.8%
Redness	2	33.4%	8	88.8%
Bleeding	5	83.4%	9	100%
Suppuration	4	66.6%	7	77.8%
Pus	1	16.7%	5	55.6%
Bacteria occurrence	5	83.3%	8	88.9%

**Table 8:** Average probing depths and peri-implantitis occurrence interval of implants of the smokers/non-smokers.

	Average Probing Depths mm ± SD	Peri-Implantitis Occurrence Interval Years ± SD	No. of Implants with Peri-Implantitis Edema and Redness
Smokers	8.30 ± 1.06	4.94 ± 3.92	2 out of 10
			χ^2^ = 7.1155, *p* = 0.007642
Non-smokers	8.25 ± 1.43	6.13 ± 5.03	13 out of 16

**Table 9:** Average probing depths and peri-implantitis occurrence interval in implants from the maxilla and mandibula.

	No. of Implants Which Have Peri-Implantitis	Average Probing Depths mm ± SD	Peri-Implantitis Occurrence Interval Years ± SD
Maxilla No. of implants 20	6 (30%)	9.17 ± 1.83	11.84 ± 2.56
	χ^2^ = 6.46,	t = 2.08046	t = 1.993
	*p* = 0.01101	*p* = 0.024164	*p* = 0.0324
Mandibula No. of implants 30	20 (66.67%)	8 ± 0.97	7.24 ± 4.84

**Table 10:** Symptom distribution between the two jaws.

	Maxilla	%	Mandibula	%
No. of implants	20		30	
No. of implants with peri-implantitis	6	30%	20	66.67%
Vertical bone loss	6	100%	20	100%
Edema	3	50%	12	60%
Redness	3	50%	12	60%
Bleeding	4	66.67%	18	90%
Suppuration	4	66.67%	17	85%
Pus	2	33.34%	6	30%
Bacteria occurrence	6	100%	15	75%

**Table 11:** Stage of implant prosthesis and peri-implantitis.

		Type of Prosthesis						
	No. Implants	Fixed Cemented FC		Fixed Screw FS		Removable Overdenture RO		FC and FS
			with PI		with PI		with PI	
Stage		16		27		4		
Type								
Prosthesis	32	16	11 68.7%	12	3 25%	4	4 100%	χ^2^ = 5.25 *p* = 0.022
PMMA Prosthesis	15			15	7 46.7%			
Healing screw	3							

**Table 12:** Average probing depths and peri-implantitis occurrence interval for different prostheses.

Average Probing Depths (mm)				Peri-Implantitis Occurrence Interval (years)			
Prosthesis			PMMA Prosthesis	Prosthesis			PMMA Prosthesis
Fixed Cemented FC	Fixed Screw FS	Removable Overdenture	Fixed Screw FS	Fixed Cemented FC	Fixed Screw FS	Removable Overdenture	Fixed Screw FS
9.09 ± 1.44	7.33 ± 0.57	8 ± 1.54	7.57± 0.54	10.63 ± 4.12	8 ± 5.29	4 (1 patient)	2 (1 patient)
FC and FS t = 2.0124, *p* = 0.03359		FC and PMMA FS t = 2.643, *p* = 0.008861		FC and RO t = 3.13, *p* = 0.003925		FC and PMMA FS t = 5.046, *p* = 0.00072	

**Table 13:** Average probing depths and peri-implantitis occurrence interval for different implant types.

Average Probing Depths mm ± SD		Peri-Implantitis Occurrence Interval Years ± SD	
Megagen AnyOne	Megagen AnyRidge	Megagen AnyOne	Megagen AnyRidge
8.11± 0.78	7.63 ± 0.91	5.66 ± 4.31	3.25 ± 1.03

**Table 14:** Percentage of implants without bacteria.

		Implants without Detection of Bacteria	
With peri-implantitis	26	5 (19.23%)	χ^2^ = 5.2653
Without peri-implantitis	24	12 (50%)	*p* = 0.021754

**Table 15:** Bacterial identification.

Bacterial Identification	No. Implants		No. Implants		No. Implants
>10^5^		10^4^–10^5^		10^3^–10^4^	
*Kocuria rosea*	2	*Staphylococcus hominis* ssp. *Hominis*	2	*Kocuria rosea*	1
*Leuconostoc mesenteroides* ssp. *Cremoris*	1	*Enterococcus faecalis*	3	*Streptococcus oralis*	1
*Streptococcus gordonii*	1	*Streptococcus anginosus*	1	*Leuconostoc mesenteroides* ssp. *Cremoris*	2
*Streptococcus constelatus*	3	*Streptococcus intermedius*	1	*Streptococcus sanguinis*	1
*Staphylococcus epidermidis*	2	*Sphingomonas paucimobilis*	1	*Staphylococcus epidermidis*	2
*Staphylococcus hominis* ssp. *Hominis*	1	*Streptococcus gordonii*	2	*Staphylococcus hominis* ssp. *Hominis*	1
*Streptococcus pseudoporcinus*	2	*Leuconostoc mesenteroides* ssp. *Cremoris*	1	*Streptococcus intermedius*	1
*Enterococcus faecalis*	3	*Kocuria rosea*	1	*Enterococcus faecalis*	1
*Yokenella regensburgei*	1			*Staphylococcus aureus*	1
*Staphylococcus aureus*	1			*Pseudomonas aeruginosa*	1

**Table 16:** Bacterial distribution by genus per number of implants.

	>10^5^	10^4^–10^5^	10^3^–10^4^	Total
Streptococcus	6	4	3	13
Staphylococcus	4	2	4	10
Enterococcus	3	3	1	7
Kocuria	2	1	1	4
Leuconostoc	1	1	2	4
Yokenella	1	0	0	1
Sphingomonas	0	1	0	1
Pseudomonas	0	0	1	1

## Data Availability

The original contributions presented in this study are included in the article/Supplementary Materials. Further inquiries can be directed to the corresponding author(s).

## Supplementary Materials

The following supporting information can be downloaded at: https://www.mdpi.com/article/10.3390/dj13040148/s1.

## Abbreviations

The following abbreviations are used in this manuscript:

PMMA	Polymethyl methacrylate
BoP	Bleeding on probing
FC	Fixed cemented
FS	Fixed screwed
RO	Removable overdenture
SD	Standard deviation
SE	Standard error
